# Robot‐assisted Percutaneous Transfacet Screw Fixation Supplementing Oblique Lateral Interbody Fusion Procedure: Accuracy and Safety Evaluation of This Novel Minimally Invasive Technique

**DOI:** 10.1111/os.12428

**Published:** 2019-02-18

**Authors:** Jing‐ye Wu, Qiang Yuan, Ya‐jun Liu, Yu‐qing Sun, Yong Zhang, Wei Tian

**Affiliations:** ^1^ Department of Spine Surgery Beijing Jishuitan Hospital Beijing China; ^2^ Beijing Key Laboratory of Robotic Orthopaedics Beijing China; ^3^ TINAVI Medical Technologies Co., Ltd Beijing China

**Keywords:** Accuracy, Lumbar disorders, Oblique lateral interbody fusion, Robot, Transfacet screw

## Abstract

**Objectives:**

Percutaneous transfacet screw fixation (pTSF) is a minimally invasive posterior fixation technique supplementing oblique lateral interbody fusion (OLIF) for lumbar spinal disorders. Accurate screw insertion is difficult to achieve and technically demanding under 2‐D fluoroscopy. Recently developed robot‐assisted spinal surgery demonstrated a high level of accuracy of pedicle screw insertion and a low complication rate. No published study has reported this combination technique. The aim of our study was to evaluate the accuracy and safety properties of the combination of both minimally invasive techniques: robot‐assisted pTSF supplementing the OLIF procedure.

**Methods:**

This was an experimental and prospective study. Selected consecutive patients with lumbar degenerative disorders received robot‐assisted pTSF supplementing the OLIF procedure using the TianJi Robot system operated by one senior surgeon from March to October 2018. The accuracy of screw insertion and perioperative screw‐related complications were evaluated. Assessment of the accuracy of screw insertion included intraoperative robotic guidance accuracy and incidence of screw encroachments. Intraoperative robotic guidance accuracy referred to translational and angular deviations of screws, which were assessed by comparing the planned and actual screw trajectories guided by the robot on reconstructed images using TianJi Robot Planning Software. Screw encroachments were evaluated on postoperative CT images and classified by a grading system (A, excellent; B, good; C, poor). Screw‐related complications including intraoperative pin skidding, screw malposition and adjustment, together with postoperative neurological symptoms that correlated with screw malposition were recorded.

**Results:**

Ten patients, with an average age of 60.2 years, were selected and recruited in this study. All cases were degenerative lumbar spinal disorders, out of which there were 6 cases of Meyerding Grade I degenerative spondylolisthesis. Twenty‐four transfacet screws were inserted by robotic assistance. Instrumented levels included nine segments at L_4–5_ level and three segments at L_3–4_ level. Two patients had both L_4–5_ and L_3–4_ level fixation. The average surgical time was 3.3 h (SD, 0.8 h). The mean blood loss was 90 mL (SD, 32 mL). Intraoperative guidance accuracy showed 1.09 ± 0.17 mm (ranging from 0.75 to 1.22 mm) translational deviation and 2.17° ± 0.39° (ranging from 1.47° to 2.54°) angular deviation. The gradings of screw encroachment were: 17 screws (71%) with Grade A, 6 screws (25%) with Grade B, and 1 screw (4%) with Grade C. Only one pin skidding occurred intraoperatively and revised subsequently. No postoperative neurological complications were found.

**Conclusion:**

Our preliminary study of robot‐assisted pTSF supplementing the OLIF procedure showed a high level of accuracy for screw insertion and this minimally invasive combination technique was found to be a feasible and safe procedure.

## Introduction

Oblique lateral interbody fusion (OLIF) is a recently developed minimally invasive surgical technique for degenerative lumbar disorders. It offers several advantages, including less bleeding^1^, achieving indirect decompression^2^, restoring high disc height^2^, ^3^, and the ability to insert a huge cage, compared with its counterpart, transforaminal lumbar interbody fusion (TLIF)^4^. Pedicle screw fixation (PSF) is the most commonly used technique in spinal surgeries including OLIF procedures. However, several drawbacks of PSF have been noticed in the literature^5^, including malposition of pedicle screws causing nerve root or spinal cord injury, and large dissection of paraspinal muscle leading to postoperative fat infiltration. Percutaneous transfacet screw fixation (pTSF) is a traditional posterior fixation method that was described several decades ago^6^, ^7^. Two cannulated cancellous screws are inserted across bilateral facet joints for each spinal segment. Translaminar transfacet screw fixation and transfacet screw fixation are two versions of pTSF. Both techniques were utilized depending on if the patient's specific anatomy allows for one technique or the other. Although translaminar transfacet screw fixation differs slightly from transfacet screw fixation, multiple studies have demonstrated that both insertion techniques lead to adequate stabilization for a successful dorsolateral spinal fusion^8^. Biomechanical studies revealed that the pTSF technique had equivalent stiffness to the PSF technique^8^, ^9^, ^10^. Meanwhile, clinical applications of the pTSF technique revealed reliable and safe results, including symptom relief and low complication rates^11^, ^12^. In a comparative and prospective study, Jang and Lee report that the clinical results of pTSF supplementing anterior lumbar interbody fusion (ALIF) were equivalent to those of the PSF supplementing technique^11^.

The conventional pTSF were performed under 2‐D fluoroscopy^13^. However, the narrow corridor of the screw trajectory cannot tolerate the minimal amount of screw deviation and revision for the misplaced screw would be quite difficult. For that reason, conventional pTSF was commonly performed under fluoroscopy by highly experienced surgeons. Even though the technique is feasible with fluoroscopic assistance, there is still high radiation exposure and high risk of malposition of the screws, which may cause decreased construct stiffness. Therefore, a high level of accuracy is required for stable and safe insertion of transfacet screws. With the advent of robot‐assisted orthopaedic surgery, robot‐assisted pedicle screw fixation had several benefits, including capability of preoperative planning, high accuracy of screw insertion, and low radiation exposure^14^. In a meta‐analysis focusing on the comparison of the accuracies of robot‐assisted and free‐hand pedicle screws insertion, 10 studies were included and analyzed. The study showed that the robot‐assisted technique is more accurate than the conventional method after analyzing the incidences of “perfect” and “clinical acceptable” pedicle screw insertions^15^.

Because precise pTSF insertion technique is demanded and the robot‐assisted surgery could provide a high level of accuracy, pTSF could be accurately performed with the assistance of robot. Theoretically, the combination of OLIF and robot‐assisted pTSF could be safely and accurately performed as a minimally invasive procedure. To our knowledge, however, robot‐assisted pTSF has not been described in the literature. We attempted to assess the safety and accuracy of this technique in a prospective and experimental study in Beijing Jishuitan Hospital. Several orthopaedic robots have been reported to be applicable for spinal procedures, including TianJi Robot, SpineAssist, Renaissance, and Mazor. SpineAssist and Renaissance, the early products of orthopaedic robots, are “bone‐mounted” machines that need temporary fixation onto the spine of interest, and synchronization of a preoperative CT scan and intraoperative fluoroscopy which is often time‐consuming is used for image registration. The TianJi Robot is an orthopaedic surgical robot developed in China with completely independent intellectual property with multiple surgical indications and wide applications for the whole spine^16^, ^17^. The TianJi Robot system with a robotic arm is not a “bone‐mounted” robot and preoperative CT scans are not required because intraoperative images obtained by C‐arm scanning are used for registration. A high level of accuracy for screw insertion and a reliable, safe process were demonstrated through the clinical use of the TianJi Robot system^18^, ^19^. During our current study, we used the TianJi Robot system to assist in the transfacet screw insertion supplementing OLIF procedure.

The aim of our preliminary study was to evaluate the safety and accuracy of robot‐assisted pTSF combined with the OLIF procedure. Both the OLIF technique and pTSF are muscle‐sparing procedures involving tiny surgical incisions. If the robot‐assisted surgery could permit a safe procedure, as a minimally invasive method, the combination method could decrease the surgical trauma to patients, with less blood loss and approach‐related musculature disruption. Therefore, it could allow for faster recovery and better patient‐reported quality of life. Compared with conventional pedicle screw fixation, pTSF only needs two cannulated screws for each segment, which are much less expensive than pedicle screws and would mean less cost for patients and society. Radiation exposure is an issue of concern during orthopaedic surgery. Conventional screw insertion is usually performed under fluoroscopy and there is a constant need for images during surgery. Robot‐assisted surgery allows for perioperative planning and single imaging may decrease the amount of radiation exposure for both patients and surgeons^20^.

## Materials and Methods

### *Patients and the Robotic System*

Selected consecutive patients were prospectively studied from March 2018 to October 2018 in Beijing Jishuitan Hospital. Patients with degenerative lumbar spine disorders, suffering from incapacitating back or radiating pain which could not be relieved by conservative treatment for more than 3 months, were included in this study. The exclusional criteria were Meyerding II degenerative spondylolisthesis or above, lumbar instability caused by neoplasm, infection or trauma, severe facet joint arthropathy, multi‐level lumbar spinal fusion, and spondylotic spondylolisthesis. Patients with neurological symptoms that could not be partially relieved by resting on a bed were also excluded. Informed consent was obtained preoperatively for the selected patients. These surgeries were performed by a senior spine surgeon (Qiang Yuan, the second author) who had the experience of more than 50 robot‐assisted spinal surgeries.

### *Introduction of the TianJi Robot System*

**Figure 1:**
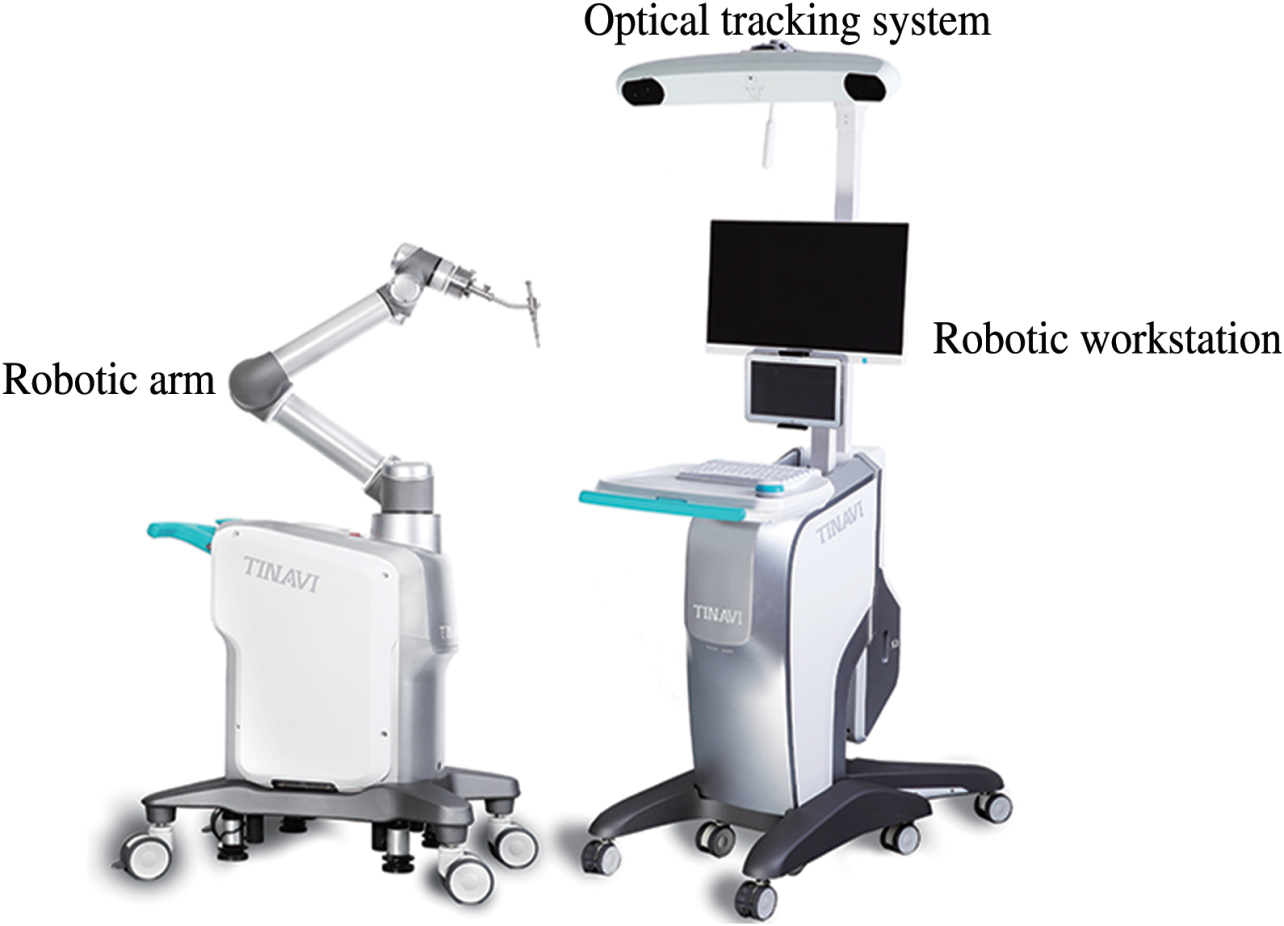
TianJi Robot system.

The TianJi Robot (Beijing Tinavi Medical Technology) is not a “bone‐mounted” robot but an image‐navigated robotic positioning platform, which comprises a robotic arm system, an optical tracking system, and a robotic workstation (including the monitor screen). During the procedure, images obtained intraoperatively by C‐arm are transferred into the robotic workstation and 3‐D images are created. Surgeons’ planning of the screw trajectories is performed on the robotic workstation. Afterwards, the robotic arm with a guidance cannula on its end automatically moves to the surgical field and guides the pin insertion within the cannula of variable inner diameter. A fluoroscopic re‐scan by C‐arm is performed and followed by cannulated or conventional screw placements if the optimal pin trajectories are confirmed (Fig. 1).

### *Surgical Techniques*

#### *Oblique Lateral Interbody Fusion Procedure*

The patient was placed at a right decubitus position on a radiolucent table. Oblique skin incision was marked within the range of 4 to 10 cm anterior to the disc or vertebral body of interest under fluoroscopic guidance. After prepping and draping, the skin and superficial layer were incised, followed by blunt dissection of abdominal muscle. The interval between the psoas major and the aorta was bluntly dissected using fingers. Afterwards, the cannulated corridor was established at the level of intervertebral space. The intervertebral disc was removed and both endplates were prepared for cage insertion. Subsequently, the cage (Clydesdale Spinal System, Medtronic, or Oracle system, Synthes) of appropriate size containing allograft was inserted and the optimal position was confirmed by fluoroscopy.

#### *Robot‐assisted Percutaneous Transfacet Screw Fixation Procedure*

The patient was positioned in a prone position after completing the OLIF procedure. The surgical field was prepped and draped. A 2‐cm small midline incision was utilized for anchoring a patient tracker onto the spinous process. The patient tracker was necessary for navigational purposes. An alternative method for patient tracker fixation is sticking the tracker onto the skin surface using several sterile drapes. Fluoroscopic images were obtained by C‐arm (ARCADIS Orbic 3D C‐arm, Siemens) and transferred to the robotic workstation. 3‐D reconstructed images were generated and displayed on the monitor screen.

**Figure 2:**
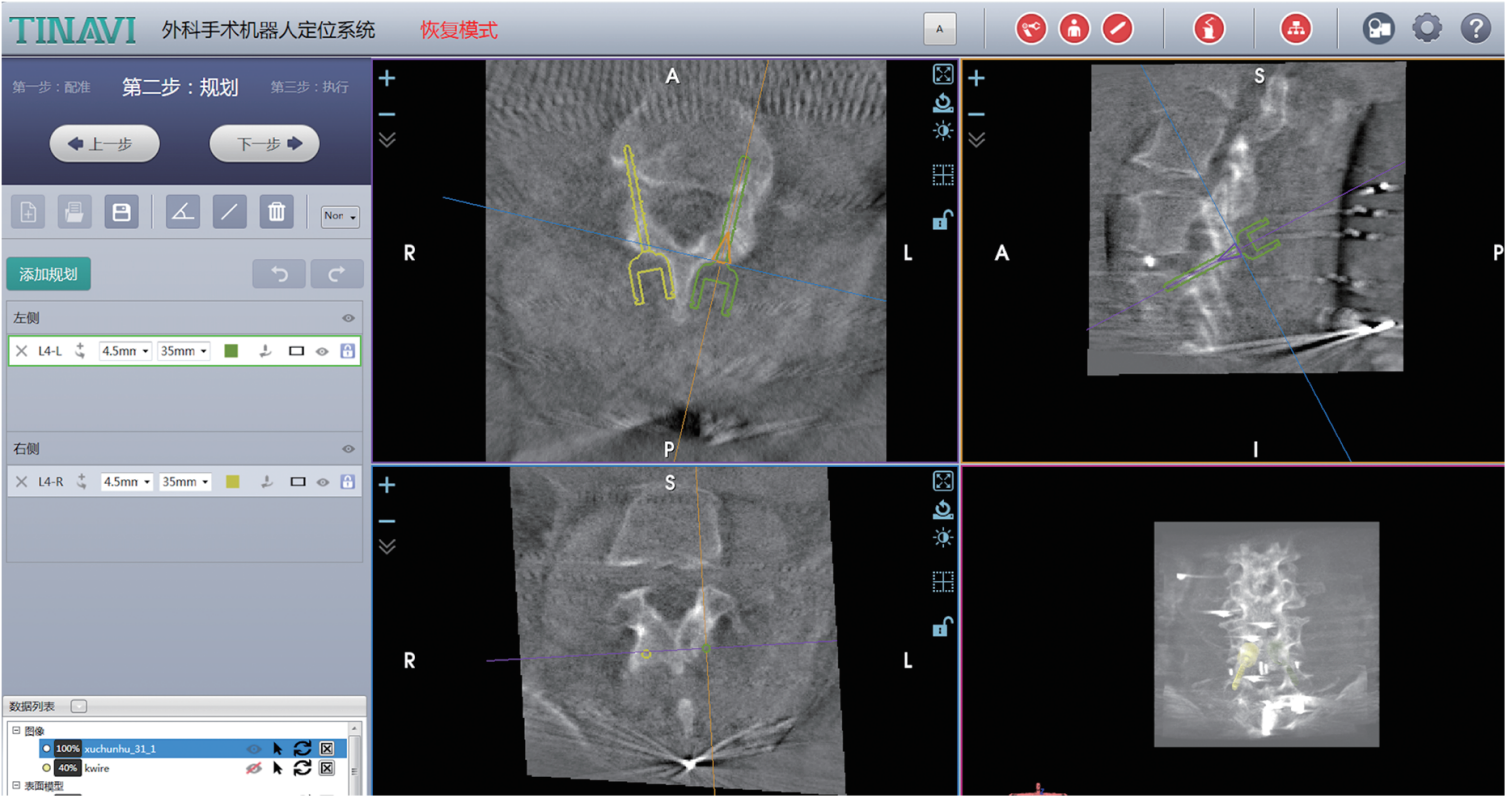
Trajectory planning on robotic workstation. Green and yellow screw represented the trajectory of transfacet screws.

The senior surgeon planned the optimal trajectory of transfacet screws on the workstation (Fig. 2). The entry point was located at the superomedial portion of the inferior articular process of the cephalad vertebra. The direction of the screw trajectory was toward the pedicle of the caudal vertebra and not beyond the bony surface. The alternative technique was translaminar transfacet screw fixation only if there was impingement of the trajectory on the midline spinous process. The entry point of the translaminar transfacet screw was located at the contralateral side of the laminar surface.

**Figure 3:**
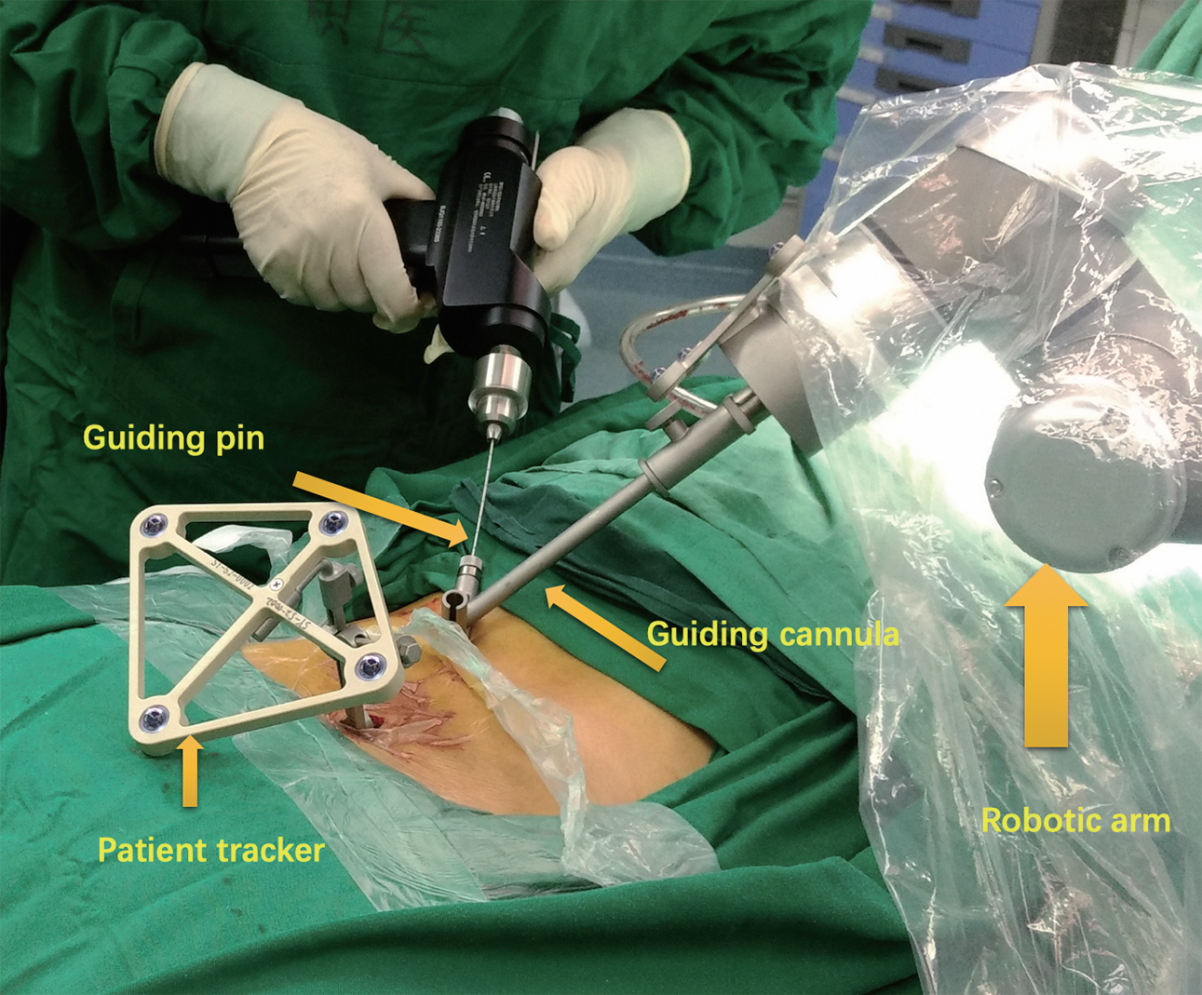
Guiding pin insertion with assistance of robot.

After finishing the planning of transfacet screws, the robotic arm was instructed to move to the surgical field. The guiding cannula was placed onto the robotic arm and approached the skin closely. The accuracy of guidance was shown on the monitor screen. Once the accuracy of guidance was less than 0.5 mm and became steady, the robot was ready to guide the pin insertion. A tiny incision was made. The guiding pin held by a drilling bit was placed along the guiding cannula at the end of the robotic arm. The pin was advanced until the bony surface was touched. The guiding pin was gently drilled to optimal depth (usually 30 mm). See Fig. 3.

**Figure 4:**
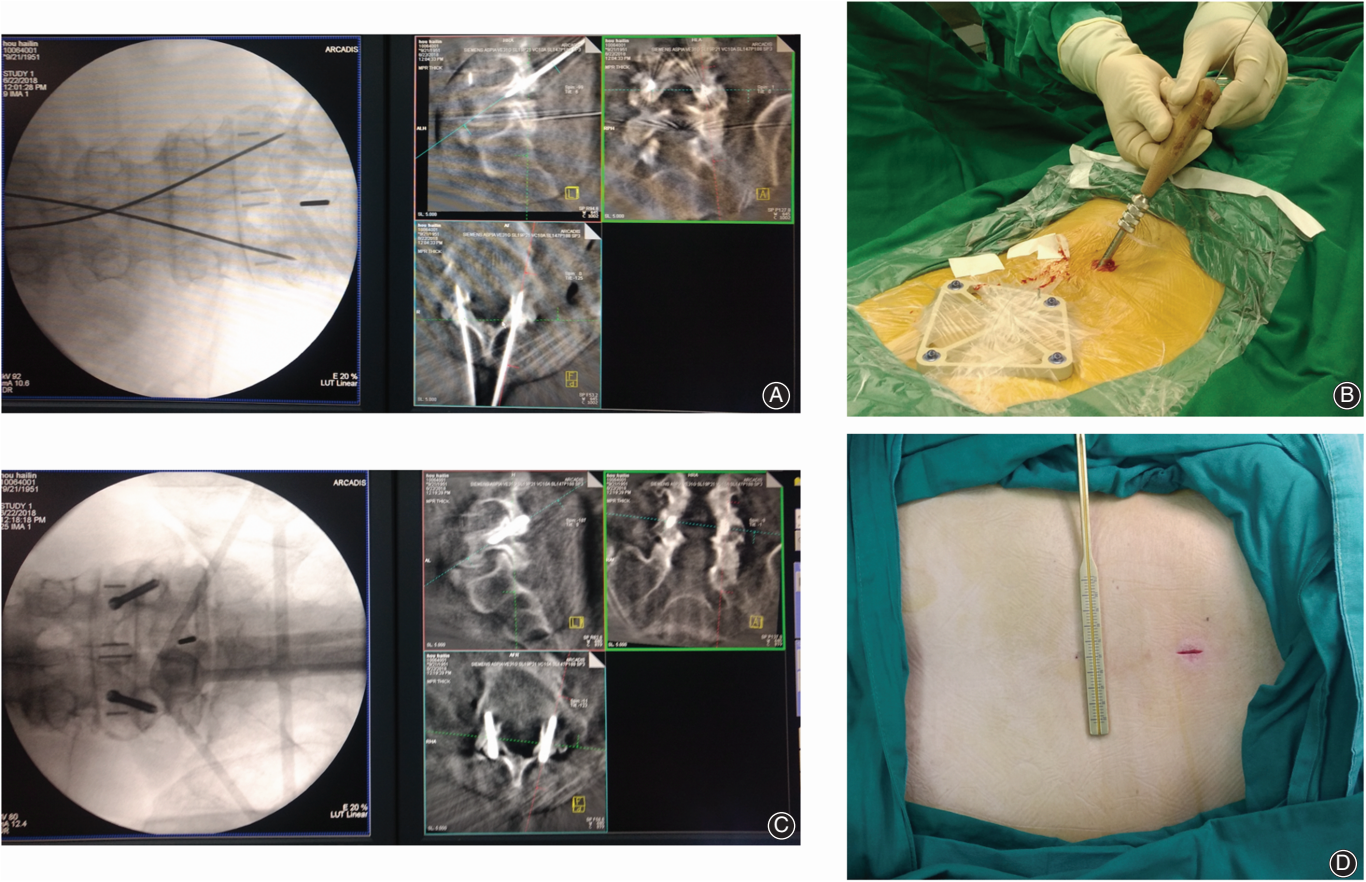
The process of transfacet screw insertion. (A) Optimal pin position confirmed by reconstructed fluoroscopic images. (B) Insertion of the cannulate AO screw along the pin guidance. (C) Screw position confirmation. (D) 1.5‐cm tiny incision at midline.

A fluoroscopic re‐scan by C‐arm was performed and the position of guiding pins was evaluated. If there is any deviation of the guiding pin, the trajectory should be revised. If the optimal position of the guiding pin was confirmed, the pilot hole was tapped, followed by insertion of cannulated Herbert screws (HCS Screw ø4.5, or Cannulated Screw ø4, DepuySynthes). Afterwards, the positions of transfacet screws were evaluated and confirmed under fluoroscopy. See Fig. 4.

### *Outcome Measures*

#### *Accuracy Assessment of Screw Insertion*

The accuracy of screw insertion was evaluated, which included intraoperative robotic guidance accuracy and grading of screw encroachment.

#### *Robotic Guidance Accuracy*

**Figure 5:**
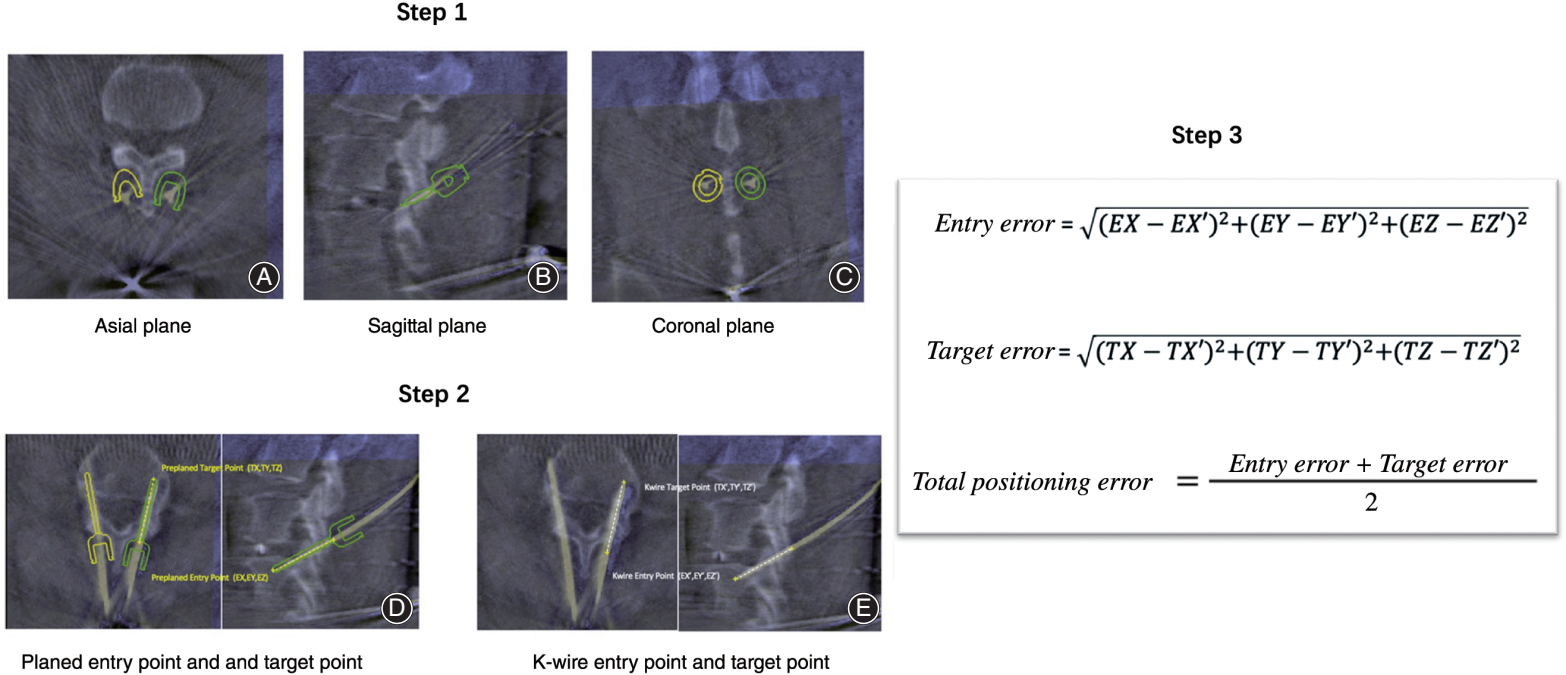
Step 1: Sample fused images, planned images as background images (color pink); K‐wire placement images as float images (color green). Bony structures aligned well in three planes: (A) Axial plane, (B) Sagittal plane, and (C) Coronal plane. Step 2: Pick up the 3‐D coordinates (EX, EY, EZ) of entry point and target point of the planned screw trajectory. Identify and pick up the 3‐D coordinates (TX′, TY′, TZ′) of the K‐wire placement: (D) Preplanned entry point and target point and (E) K‐wire entry point and target point. Step 3: Euclidean distance was used to calculate this deviation.

Robotic guidance accuracy refers to the degree of translational and angular deviations during the robotic guiding process. Measurements were used to compare the planned and actual trajectories on reconstructed images obtained by C‐arm (called CBCT). To enable a comparison in the same plane, CBCT‐to‐CBCT overlay software (TianJi Robot Planning Software) was used to fuse each patient's two 3‐D fluoroscopic scans. Methods of measurements are listed below and illustrated on Fig. 5.

- Step 1: Image fusions. Using TianJi Robot Spine Software to fuse planned CBCT and K‐wire placement CBCT into a single image.
- Step 2: Entry and target point recognition. Identify the entry and target point of the planned screw trajectory and K‐wire placement, respectively.
- Step 3: Calculate the deviation between planned trajectory and K‐wire placement. Euclidean distance was used to calculate the displacement deviations.
- Step 4: Measure the angular deviations in the axial and sagittal plane, respectively. Calculate the average value of both angular deviations in the dual planes.

#### *Grading of Screw Encroachment*

**Figure 6:**
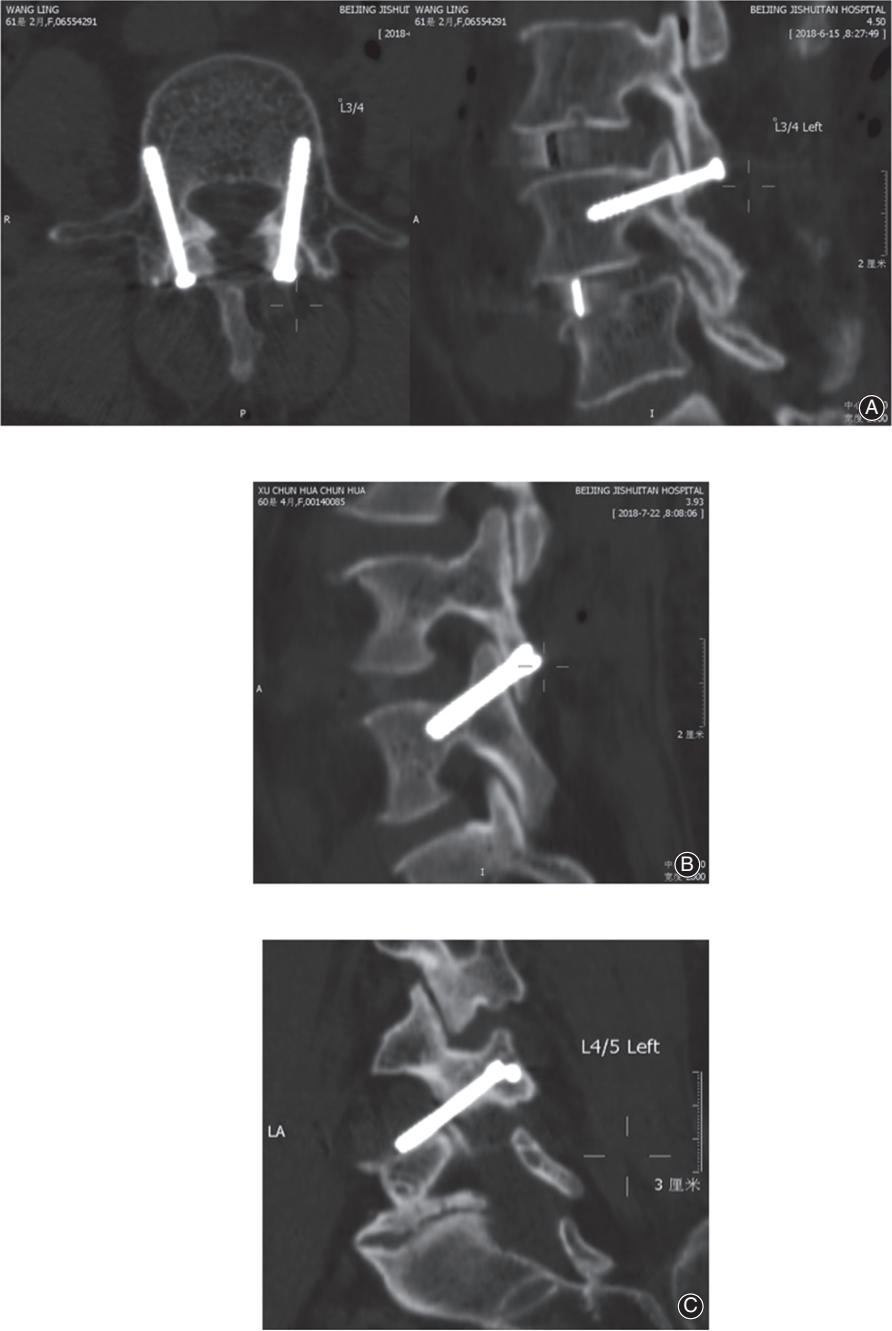
Grading system for screw positions: (A) Grade A, no cortex perforation; (B) Grade B, cortex perforation within 2 mm; and (C) Grade C, cortex perforation beyond 2 mm.

Postoperative screw encroachment of the cortex was evaluated on the postoperative CT scans. Positions of screws were qualified and graded (Fig. 6). Grade A, the excellent screw position, refers to no occurrence of cortex perforation. Grade B, the good screw position, refers to cortex perforation within 2 mm. Grade C, the poor screw position, refers to cortex perforation beyond 2 mm. The qualification of screw placement was evaluated and graded by two observers (the first and second author). The lower grade for each screw was chosen in two observers’ results.

#### *Evaluation of Intraoperative and Early Postoperative Complications*

The intraoperative and recent postoperative complications related to screw malposition were recorded. The intraoperative complications include technical issues, such as hardwire failure and K‐wire skidding causing screw misplacements. Postoperative complications include neurological compromise, such as radiating pain, numbness, and decreased muscle power, which correlate with screw malposition. Other early postoperative complications irrelevant to screw malposition were also recorded.

### *Statistical Analysis*

The quantitative and qualitative data was collected and analyzed using Microsoft Excel (Version 16.16).

## Results

### *General Information*

Ten patients were prospectively studied, out of which 3 were men and 7 women. The average age was 60.2 years (SD, 7.6). All cases were degenerative lumbar spinal disorders, out of which there were 6 cases of Meyerding Grade I degenerative spondylolisthesis and 4 cases of lumbar segmental instability and lumbar stenosis. One patient had previous L_4–5_ instrumentation and fusion. Detailed information for each case is shown on Table 1.

**Table 1:** Detailed information for each case

Case	Sex	Age (years)	Diagnosis	Segment	Transfacet screw fixation technique	Numbers of screws	Surgical time (h)	Blood loss (mL)	Intraoperative complications
1	F	53	Degenerative spondylolisthesis	L_4–5_	Translaminar	2	3.5	50	None
2	F	51	Degenerative spondylolisthesis	L_4–5_	Translaminar	2	4.5	100	None
3	F	53	Lumbar segmental instability	L_3–4_	Translaminar	4	3.5	100	None
4	F	61	Degenerative spondylolisthesis	L_3–4_ & L_4–5_	Transfacet	2	4.5	150	None
5	M	67	Degenerative spondylolisthesis	L_4–5_	Transfacet	2	3	100	None
6	M	73	Degenerative spondylolisthesis	L_4–5_	Translaminar	2	2	100	K‐wire skidding
7	F	60	Degenerative spinal stenosis	L_4–5_	Transfacet	2	3.5	50	None
8	F	61	Degenerative spondylolisthesis	L_3–4_ & L_4–5_	Transfacet	2	3	100	None
9	M	54	Degenerative spinal stenosis	L_4–5_	Transfacet	2	3	50	None
10	F	69	Degenerative spinal stenosis	L_4–5_	Transfacet	4	2.5	100	None

F, female; M, male.

### *Operation Information*

Instrumented levels included nine segments at L_4–5_ level and three segments at L_3–4_ level. Two patients had both L_4–5_ and L_3–4_ level fixation. Twenty‐four transfacet screws were inserted. Ten screws were inserted as translaminar transfacet screws, while the others were transfacet screws. The average surgical time was 3.3 h (SD, 0.8 h). The mean blood loss was 90 mL (SD, 32 mL).

### *Accuracy and Complication Evaluation*

The average displacement and angular deviations of these 24 screws were 1.09 ± 0.17 mm (ranging from 0.75 to 1.22 mm) and 2.17° ± 0.39° (ranging from 1.47° to 2.54°), respectively. The gradings of screw encroachment were 17 screws (71%) of Grade A, 6 screws (25%) of Grade B, and 1 screw (4%) of Grade C.

During the procedure, only 1 case had K‐wire skidding in the drilling holes at the cortex and the trajectory was revised after the fluoroscopic confirmation. No screw misplacement was noticed during the procedure. Five patients had numbness over the anterior thigh and iliopsoas muscle weakness, which subsided within 2 weeks. No complications were found to be correlated with the screw malposition.

## Discussion

The oblique lateral approach is a muscle‐sparing route for cage insertion and the cages used in the OLIF procedure are larger than the counterparts used in the TLIF procedure, which could improve the lumbar alignment to a greater extent^4^. Due to insertion of the large cage, the height of disc space is restored, which results in shrinkage of the bulging disc and flattening of the folded ligamentum flavum. Therefore, these improvements could increase the dimensions of the vertebral canal and the lateral recess. These findings demonstrate how indirect decompression can be achieved and that percutaneous posterior fixation can be performed without direct decompression.

Posterior fixation is typically required in OLIF procedures to reinforce the segmental stability. As one stability supplement method, pTSF has several advantages compared with open TLIF surgeries: there are few incisions, there is minimal blood loss, and only two screws are used each spinal segment, which are less expensive than pedicle screws for. In our study, only one tiny incision was needed for pTSF in several selected patients and each case only had 90 mL of blood loss on average. Those findings suggest that this combination technique is a promising minimally invasive procedure.

### *Application of Percutaneous Transfacet Screw Fixation Technique*

Transfacet screw fixation in the lumbar spine was described by King^6^ and Boucher^7^ in the 1940–1950s. The stability of pTSF is still an issue of concern for some surgeons. However, biomechanical research revealed that pTSF had equivalent stiffness against segmental movements compared with PSF. Ferrara *et al*. report a cadaveric study showing the same stiffness between pTSF and PSF techniques in the short‐term phase and the long‐term cyclic loading phase^9^. The study performed by Chin *et al*. even shows that pTSF has greater stability than pedicle screws in motion of flexion^10^.

The clinical results of pTSF as a minimally invasive posterior fixation technique are examined in several published studies and promising results are reported in the literature^11^, ^12^, ^21^, ^22^. Felbaum *et al*. performed a retrospective study evaluating the accuracy, hardware failure, and complications of pTSF in ALIF and lateral lumbar interbody fusion (LLIF)^21^. The results showed that pTSF was a safe and reliable procedure. Rhee *et al*. evaluated the long‐term results of LLIF supplemented with pTSF^22^. Eighty‐nine percent of patients had symptomatic relief and 72% of patients had good to excellent results. All the patients had solid fusion at 1‐year follow‐up.

2‐D fluoroscopic guidance is the method most commonly used to assist in transfacet screw insertion^13^, ^23^, ^24^, ^25^. However, the accuracy and reliability of this form of guidance is still a concern. Even with a guide device and fluoroscopic assistance, Jang *et al*. reported that the perforation rate of the transfacet screw was 34%^26^. Shim *et al*. reported the clinical results of fluoroscopy‐assisted transfacet screw insertion^25^. In their study, 15.4% of transfacet screws failed to purchase the pedicle, although there was no detailed grading information of the screw trajectory. The amount of bone purchase for each transfacet screws accounts for the stability of the construct. To achieve reliable fixation, surgeons try to find an optimal pathway which usually has a narrow corridor for the trajectory of transfect screws. Good interpretation of fluoroscopy and anatomy, together with surgical experience are necessary for accurate insertion under fluoroscopy. However, prolonged exposure to radiation is inevitable with repeated fluoroscopy for screw adjustment or confirmation.

### *Accuracy and Safety Evaluation*

Robot‐assisted spinal surgery showed high accuracy of screw placement: 92.9% to 100% accuracy of pedicle screws was reported using Gertzbein and Robbins criteria in analyzing screw placement accuracy^14^. In an RCT study comparing robot‐assisted and open free‐hand lumbar instrumentation surgeries with recruitment of 60 patients, Hyun *et al*. found that the breach incidence of pedicle screws was lower in the robot‐assisted group and breaches only occurred in the lateral direction, compared with medial or inferior breaches in free‐hand group^20^. No published studies report the accuracy of robot‐assisted pTSF procure. Our study provides accurate data for this technique. The robot guidance accuracy was approximately 1 mm with 2° deviations, which were acceptable accuracy requirements in the setting of the pTSF technique. Postoperative CT assessment of transfacet screws showed 96% excellent to good screw positions. These finding show that this combination technique of OLIF and robot‐assisted pTSF is feasible and reliable in clinical practice.

With respect to the safety of this technique, intraoperative and early postoperative complications of robot‐assisted pTSF were evaluated in our study. Only 1 case had K‐wire skidding, which was revised afterwards. Early postoperative complications showed that 5 cases had OLIF approach‐related complications, including numbness over the anterior thigh and iliopsoas weakness. No complications were found to correlate with screw malposition. These results demonstrate that this technique is a safe procedure.

Pin skidding during drilling and the insertion process was the most common intraoperative complication in percutaneous robot‐assisted procedures^14^. To avoid skidding on the cortex of lamina, there are several precautions to be noted based on our experience. The diameters of the sleeve and the guiding pin should be closely matched. Any spaces between these two devices would result in pin deviations during drilling. Besides, the drill bit to facilitate the pin advancement was necessary. Low‐speed drilling at the initial stage could create an indentation on the cortex, which can permit further guiding pin advancement without skidding and, subsequently, the guiding pin can be drilled at high speed to an appropriate depth.

### *Limitations of the Study*

There are several limitations of our study. The sample size of patients was small in this preliminary study and the number of cases would be increased in our future study. In future study, the mid‐term and long‐term clinical results of this technique will be evaluated during the patients’ follow‐up.
